# Incidence of calcaneal apophysitis in Northwest Istanbul

**DOI:** 10.1186/s12891-018-2184-6

**Published:** 2018-07-27

**Authors:** H. H. Ceylan, B. Caypinar

**Affiliations:** 1 Lutfiye Nuri Burat Devlet Hastanesi, 50.Yil Mah., 2107 Sok, 34256 Sultangazi, Istanbul, Turkey; 2 Gelisim University, Istanbul, Turkey

**Keywords:** Sever’s disease, Calcaneal apophysitis, Heel pain

## Abstract

**Background:**

Calcaneal apophysitis is a common clinical entity affecting children and adolescents. It is also known as Sever’s disease. Heel pain without a recent trauma is the primary manifestation. There are limited studies on the incidence of this disease. In this study, we aimed to report the regional incidence in Istanbul.

**Methods:**

This retrospective audit of health records of all paediatric patients aged 6–17 years between January 1, 2014, and December 15, 2017 was undertaken. During this period, data were extracted from health records that recorded calcaneal apophysitis as the primary diagnosis.

**Results:**

The 4-year incidence of calcaneal apophysitis was found to be 0.35% (74 of 20,967 paediatric patients). It commonly affected males, and bilateral cases were more common than unilateral cases. There were more admissions during the spring season, which may indicate a possible association with physical activity.

**Conclusion:**

Although calcaneal apophysitis is a relatively common paediatric foot problem, due to its benign course and spontaneous healing capacity, most physicians are not interested in this topic. However, increased awareness of this diagnosis is important for reducing the rates of unnecessary radiological examinations and orthopaedic referrals. With increased knowledge, most cases may be diagnosed at the family physician level, which may decrease the economic burden on the health system. Incidence reports from various countries and regions may be published in the future.

## Background

Calcaneal apophysitis, or Sever’s disease, is a common entity among paediatric and adolescent patients who present to a physician with heel pain [1–11]. This disorder is an overuse syndrome and was first identified by James Warren Sever in 1912 [12]. The basic pathology is repetitive microtrauma that induces calcaneal apophysis damage [7, 13, 14]. Although various mechanisms have been discussed in the literature, there is not a single mechanism explaining this pathology in all cases. The clinical manifestation is typically characterised by pain that can be localised by palpation to the posteroinferior region of the calcaneus. The medial-lateral squeeze test may be helpful for clinical diagnosis. The symptoms are exacerbated by sports that require extreme physical activity [1, 5–7, 11, 13, 15, 16]. Although the symptoms are usually associated with physical activity, resting pain can also be observed in advanced cases. Different studies have shown the considerable impact of calcaneal apophysitis on health-related quality of life [9, 17].

Calcaneal apophysitis tends to occur between the ages of 8 and 13 years in girls and between the ages of 11 and 15 years in boys [7]. Its incidence among all musculoskeletal injuries has been reported to be between 2 and 16%, [1] but is believed to be even higher in the active paediatric population [1, 5–7, 11, 15, 16]. Calcaneal apophysitis has a benign course, and treatment is conservative. Resting, applying ice, stretching, strengthening the calf muscles, using heel-elevating supports or orthoses and taking anti-inflammatory drugs largely solve the problem [1, 2, 7, 10, 13, 18–21]. Fixation with a resting plaster has been reported for rare, persistent cases [6, 13]. Although the disease usually has a benign course, it sometimes requires extended treatment or causes an active athlete to stay away from the field for a period of time [7, 14]. Anamnesis and physical examination are sufficient for diagnosis [1, 3, 7, 8, 10–12, 22, 23]. Magnetic resonance imaging is recommended for ruling out fracture, tumour, or infection in suspicious cases [24].

Increased knowledge of calcaneal apophysitis and its incidence will help physicians diagnose the disease with anamnesis and examination and help reduce the need for further radiological evaluations, which are harmful to patients. In this study, we aimed to determine the incidence of calcaneal apophysitis by examining the data on paediatric patients with heel pain who were admitted to our hospital located in Northwest Istanbul, which has a higher birth-rate and larger paediatric population than most regional hospitals of Turkey.

## Methods

Paediatric patients who were admitted to our outpatient clinic with heel pain over 4 years (between January 1, 2014, to December 15, 2017) and diagnosed with calcaneal apophysitis were included in the study.

The records of all patients who were admitted to our outpatient clinic within these 4 years and aged between 6 and 17 years at the time of initial admission were obtained from the database records. Both orthopaedic surgeons had a standard approach to paediatric heel pain cases, which included a detailed anamnesis and physical examination. In addition to the detailed anamnesis, the heel squeeze test was applied for all children bilaterally and recorded in the patient’s initial visit. The anamnesis records were scanned for the terms *‘heel’* and *‘Sever’*. All accessed files were transferred to a table sheet, and content verification was conducted on individual records separately by two orthopaedic surgeons. At first, all trauma cases were excluded. Second, files without a diagnosis of calcaneal apophysitis or those including an anamnesis record that differed from the manifestation of calcaneal apophysitis were excluded. Among the remaining files, patients with missing or incomplete anamnesis data (*n* = 12) or patients whose diagnosis of calcaneal apophysitis seemed suspicious (*n* = 5) were also excluded. In all, a total of 74 patients were identified. The patients’ age, sex, affected side, admission month, and symptom duration before admission were noted.

## Results

During this four-year period, a total of 20,967 paediatric patients aged 6 to 17 years were admitted to our hospital for various complaints. Only 91 of them had a complaint of isolated calcaneal tenderness without a history of recent trauma. Among these 91 children, 74 were identified to have a diagnosis of calcaneal apophysitis.

The anamnesis records revealed that the distribution of patients who had a diagnosis of calcaneal apophysitis changed each year. The case distribution in the last 4 years was noted: there were 23 cases in 2014, 19 in 2015, 11 in 2016, and 21 in 2017 (Table 1). Of the 74 patients diagnosed with calcaneal apophysitis, 59 were male, and 15 were female. The mean age of our patient group was 10.77 (6.87–15.73) years. The average age was 11.14 (8.04–15.73) years for boys and 9.28 (6.87–13.20) years for girls at the time of each admission. Of the 74 patients, symptoms were bilateral in 46 (62.16%) patients and unilateral in the rest. The average time between the onset of complaints and admission to the outpatient clinic was 12.7 (min 2-max 108) weeks. The patient with the longest period of complaints was a girl who was 11 years old at the time of admission.

**Table 1:** Distribution of cases for each year

Year	Diagnosis of Sever’s disease (n)	Total number of admissions aged 6–17 years (n)	Incidence (%)
2014	23	5111	0.45
2015	19	4819	0.39
2016	11	4519	0.24
2017	21	6518	0.32
Overall	74	20,967	0.35

At first, ibuprofen treatment was initiated in all patients. The daily dose was divided into two and suggested to be given every 12 h. Additionally, stretching exercises were described to the families, and they were asked to monitor the child’s practice at home. All patients were called for follow-up after 2 weeks of treatment. Among the 69 patients who were reached for follow-up, the treatment was found to be effective. The exception was a 9-year-old boy. An MRI examination was obtained to exclude other possible pathologies but revealed no additional pathology except oedema of the calcaneal apophysis. The patient underwent non-weight-bearing mobilisation with a custom-made ankle orthosis for 4 weeks. Then, due to residual pain and dissatisfaction, passive stretching exercises for 2 weeks under a physiotherapist’s supervision were attempted. Although the patient still reported some residual pain in the eighth week after the treatment was completed, his family declared that the symptoms were significantly relieved compared to those at the first admission. X-ray examination was performed for all of our patients upon request of their families.

**Fig. 1:**
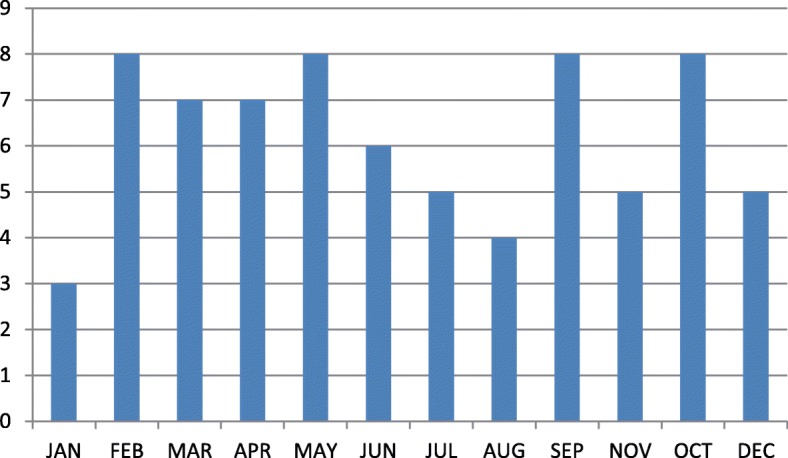
Distribution of all calcaneal apophysitis cases upon admission

The 4-year incidence of calcaneal apophysitis was found to be 0.35% (74 of 20,967). Calcaneal apophysitis commonly affected males, and bilateral involvement was more common than unilateral involvement. We observed that there were more admissions during the spring season (Fig. 1). This finding indicates a possible association of the disease with physical activity.

## Discussion

In this study, we aimed to determine the incidence of calcaneal apophysitis in the general paediatric population from records at our hospital. Due to the higher birth-rate and higher paediatric patient admission rates at our hospital than at most regional hospitals of Turkey, the reported data are able to reflect the actual disease incidence in the general population. The large sample size is an advantage of our study, as it reduces the risk of detecting coincidentally high incidences. The overall 4-year incidence of calcaneal apophysitis was found to be 0.35% in our cohort. Boys were affected more often than girls, and bilateral presentation was dominant.

Although calcaneal apophysitis is common in paediatric patients with heel pain, there are limited studies on the incidence and prevalence of the disease in the general population [11]. Sports trauma clinics have reported higher incidences, but these data were considered insufficient for reflecting the overall incidence [20]. Orava reported the 6-years incidence of calcaneal apophysitis to be 22.7% [16]. These previous studies focused primarily on patient cohorts, which may be considered an overuse group and may be biased when representing the actual incidence of calcaneal apophysitis [11]. In a study by Wiegerinck et al. in the Netherlands, the authors evaluated general paediatric population data over three consecutive years, they calculated the annual incidence rate and then calculate the mean over the 3 years, and reported the 3-years incidence of calcaneal apophysitis to be 0.37%, which is similar to our findings [11]. In the same study, the authors reported the disease’s annual incidence in 2010 to be 0.49% based on physicians’ records [11]. However, it was also emphasised that this increase in disease incidence may be entirely incidental. Similar to the mentioned study, the annual incidence changed year by year in our cohort, and we found the 4-year incidence of calcaneal apophysitis in the general population to be approximately 0.35%.

Calcaneal apophysitis was radiologically and clinically identified by Sever in the early twentieth century and was called Sever’s disease in the literature [12]. Sever’s first definition reported that this disease occurred primarily in inactive and overweight children [12]. Radiological findings related to the disease were also emphasised in this first report. Lewin claimed that this condition was a painful inflammation of the epiphysis and was a result of traction of the epiphysis by the Achilles tendon and plantar fascia in opposite directions [22]. High levels of activity and obesity were identified as risk factors [14, 25]. Apophyseal traction in the insertion side of the Achilles tendon may be related to overuse during the rapid growth period in adolescence [23]. Calcaneal apophysitis has a benign course that is self-limiting in nature [16]. Symptoms typically resolve after fusion of the apophysis and calcaneus [12]. The inflammatory process rarely results in an apophyseal fracture, [26] and no apophyseal fractures were found in our patient group.

Calcaneal apophysitis typically presents in children between the ages of 8 and 15 years [2, 23]. A case of calcaneal apophysitis in a 6 year old was reported in the literature [27]. This condition is 2–3 times more common in males than in females, and the symptoms are bilateral in 60% of cases [4]. In accordance with current knowledge, the age distribution of our patient group is between 6 and 15 years old, and boys were affected more often than were girls. The symptoms were bilateral in 62.16% of patients.

A conservative approach is commonly adopted in the treatment of calcaneal apophysitis. Ice application, stretching, resting and activity modification are treatment methods emphasised in the literature [3, 5, 14, 15, 21]. Studies that reported the positive effects of heel supports, ice application, and stretching used these methods in combination [5, 7, 21]. Similarly, some authors recommended heel supports in addition to restrictions on sports activity [1, 10, 18]. Some authors recommended using arch-supporting devices, which increase the traction effect of the gastrocnemius-soleus complex on pronating deformities of the foot [14]. Heel supports are thought to be effective through decreasing the traction effect of the gastrocnemius-soleus complex on the growing apophysis [28, 29]. The best method for calcaneal apophysitis management is not clear, and definitive evidence of the superiority of these known methods is limited [1, 14]. Wiegerinck et al. compared the wait-and-see, heel raise inlay, and physical therapy methods and could not find a significant difference among these methods [30]. However, the heel support group was more satisfied with the outcomes than the other groups after 6 weeks of treatment. A current prospective randomised study by James et al. showed the positive effect of heel risers over prefabricated orthotics in the early period of the disease [14]. In our patient group, none of the patients were recommended the use of heel or arch supports. Only one resistant case required fixation with an orthosis and non-weight-bearing mobilisation for 4 weeks.

There is limited literature on the efficacy of nonsteroidal anti-inflammatory therapy in the treatment of calcaneal apophysitis. Karahan et al. reported good results with 3 weeks of ibuprofen and topical diclofenac in addition to heel supports and stretching exercises [31]. Oral NSAIDs and short leg fixation plasters [6, 24] and local ketoprofen gel administration were reported in other studies [21]. We prescribed appropriate doses of ibuprofen to all our patients and found it to be a cheap and effective treatment.

Increased calcaneal apophysis density and fragmentation are observed on direct X-ray evaluation in calcaneal apophysitis. However, these findings are not pathognomonic to calcaneal apophysitis and may also be observed in healthy children [1, 3, 7, 8, 10, 16, 23, 24, 27, 31]. While the diagnosis is based on the clinical presentation and anamnesis, direct X-ray may be used to exclude other potential pathologies [24]. Possible reasons for heel pain, such as stress fracture, osteomyelitis, Achilles tendinitis, and calcaneal cysts, should be considered in the differential diagnosis [24]. An MRI examination may be useful for this purpose. In calcaneal apophysitis, MRI findings are limited to bone marrow oedema in most cases, and increased gadolinium uptake may be another finding [24]. In our patient group, we did not use such imaging methods, except in one patient that underwent an MRI scan due to persistent pain. A single lateral calcaneus X-ray image was obtained from all patients for confirmation only.

This study has certain limitations. Due to the retrospective nature of this study, some of the calcaneal apophysitis cases in our hospital database may have been excluded due to the inability to access their records or incomplete anamnesis forms completed during admission. As emphasised in a previous study, 50% of patients with musculoskeletal disorders do not consult a physician for their complaints [32]. Some calcaneal apophysitis patients may go to their local doctor, physical therapist or podiatrist, and some may never be admitted to a hospital despite their complaints and ability to reach the hospital. These patients may have received treatment from their family physicians without a diagnosis. For these reasons, the actual incidence may be higher than that indicated by our study. The second limitation was that X-ray examination was performed upon the request of the families. Because parents are paying for their child’s healthcare, and want an X-ray to be taken. X-ray examination is not necessary, and the current literature strongly advises against taking an X-ray for calcaneal apophysitis diagnosis. However, socio-economic conditions forced us to use X-ray examination. Although we could not detect any atypical findings, one of our patients was resistant to medical treatment. We also used non-weight mobilisation based on outdated literature, which was the wrong approach based on current knowledge. Restriction of daily activities is not currently recommended. Another limitation is that there were no available data on the sports or daily activity levels of the children at initial admission. Therefore, these details could not be discussed in our study. A prospective study can record complete patient data and include records from the family physician, podiatrist, and orthopaedic surgeon, thus overcoming these obstacles and allowing a more precise regional incidence to be reported.

## Conclusion

Although it is a relatively common paediatric foot problem, due to its benign course and spontaneous healing capacity, most physicians are not interested in calcaneal apophysitis. However, increased awareness of the clinical diagnosis is important for reducing the rates of unnecessary radiological examinations and orthopaedic referrals. Most cases of calcaneal apophysitis can be diagnosed by family and local physicians, which may decrease the economic burden on the health system. Further research on diagnosis and treatment needs to be published for an increased understanding of calcaneal apophysitis. In addition, further research may result in incidence reports from different regions around the world.

### Conclusion for families

When to worry? Calcaneal apophysitis is a benign and transient condition that can be easily managed at home and does not require consultation or investigation. Despite its benign course, families should be aware of some clinical signs that may indicate the presence of other heel problems. If the heel pain is constant or occurs at night, if there is erythema or swelling, if the child is unwell, and if the child is between the ages of 7–14 years, consultation with a healthcare practitioner is mandatory.

What to do? If the diagnosis is calcaneal apophysitis, do not worry. It is a transient condition related to your child’s bone growth. During this period, rest and ice application may be helpful. Some modifications to sport activities can also be effective. Shoe modification, including heel risers, may be helpful during this period. Medical treatment with basic anti-inflammatory drugs is sufficient in most cases.
